# An MRTF-A–ZEB1–IRF9 axis contributes to fibroblast–myofibroblast transition and renal fibrosis

**DOI:** 10.1038/s12276-023-00990-6

**Published:** 2023-05-01

**Authors:** Qianwen Zhao, Tinghui Shao, Yuwen Zhu, Gengjie Zong, Junjie Zhang, Shifan Tang, Yanshan Lin, Hongzhen Ma, Zhifan Jiang, Yong Xu, Xiaoyan Wu, Tao Zhang

**Affiliations:** 1 Key Laboratory of Targeted Intervention of Cardiovascular Disease and Collaborative Innovation Center for Cardiovascular Translational Medicine, Department of Pathophysiology, Nanjing Medical University, Nanjing, China; 2 Department of Geriatric Nephrology, First Affiliated Hospital to Nanjing Medical University, Nanjing, China; 3 State Key Laboratory of Natural Medicines, Department of Pharmacology, China Pharmaceutical University, Nanjing, China; 4 School of Sports and Health, Nanjing Sport Institute, Nanjing, China

**Keywords:** Transcription, Pathogenesis, Kidney diseases

## Abstract

Myofibroblasts, characterized by the expression of the matricellular protein periostin (*Postn*), mediate the profibrogenic response during tissue repair and remodeling. Previous studies have demonstrated that systemic deficiency in myocardin-related transcription factor A (MRTF-A) attenuates renal fibrosis in mice. In the present study, we investigated the myofibroblast-specific role of MRTF-A in renal fibrosis and the underlying mechanism. We report that myofibroblast-specific deletion of MRTF-A, achieved through crossbreeding *Mrtfa*-flox mice with *Postn*-Cre^ERT2^ mice, led to amelioration of renal fibrosis. RNA-seq identified zinc finger E-Box binding homeobox 1 (Zeb1) as a downstream target of MRTF-A in renal fibroblasts. MRTF-A interacts with TEA domain transcription factor 1 (TEAD1) to bind to the Zeb1 promoter and activate Zeb1 transcription. Zeb1 knockdown retarded the fibroblast–myofibroblast transition (FMyT) in vitro and dampened renal fibrosis in mice. Transcriptomic assays showed that Zeb1 might contribute to FMyT by repressing the transcription of interferon regulatory factor 9 (IRF9). IRF9 knockdown overcame the effect of Zeb1 depletion and promoted FMyT, whereas IRF9 overexpression antagonized TGF-β-induced FMyT. In conclusion, our data unveil a novel MRTF-A–Zeb1–IRF9 axis that can potentially contribute to fibroblast–myofibroblast transition and renal fibrosis. Screening for small-molecule compounds that target this axis may yield therapeutic options for the mollification of renal fibrosis.

## Kidney disease: Pathway to potential treatments for renal fibrosis

Targeting a signalling pathway involving several proteins could help treat renal fibrosis in chronic kidney disease. Renal fibrosis, regulated by a network of transcription factors, involves the accumulation of extracellular matrix proteins in the kidneys and significantly affects kidney function and prognosis of kidney diseases. Previous studies have revealed that loss of myocardin-related transcription factor A (MRTF-A) reduces renal fibrosis. Investigators in China led by Tao Zhang, Nanjing Medical University, and Xiaoyan Wu, Nanjing Sports Institute, explored the specific role of MRFT-A in myofibroblasts. Using a novel transgenic mouse strain in which MRTF-A was deleted from myofibroblasts, these investigators found that MRTF-A might rely on a transcriptional cascade consisting of ZEB1 and IRF9 to drive renal fibrosis. Targeting individual components of this axis may yield novel therapeutics for the treatment of renal fibrosis.

## Introduction

Chronic kidney disease (CKD), characterized by slow but irreversible loss of nephrons and thus renal function, currently affects approximately one out of ten adults worldwide^1^. Defined as an estimated glomerular filtration rate (eGFR) below 60 ml per min per 1.73m^2^, CKD is projected to become the fifth leading cause of death by 2040. Newly diagnosed cases of CKD have risen dramatically in recent years, largely owing to the global pandemic of metabolic syndrome and aging. Regardless of etiology, renal fibrosis, characterized by excessive deposition of extracellular matrix proteins in the interstitial space, is a hallmark feature of CKD^2^. Renal fibrosis is associated with disruption of kidney anatomy and function and inversely correlated with prognosis in CKD patients^3^. Therefore, renal fibrosis is often considered the end-point guiding the development of interventional strategies.

A specialized cell type, myofibroblasts, which emerge only after renal injury, is considered the effector of renal fibrosis^4^. The origin from which myofibroblasts are derived in fibrotic kidneys has been controversial. Tubular epithelial cells were believed to be the predominant source from which myofibroblasts arise during renal fibrosis, likely through a process known as epithelial-mesenchymal transition (EMT), but this notion has been largely debunked^5^. State-of-the-art techniques such as fate-mapping have greatly facilitated the delineation of the myofibroblast lineage, but the results are often subjected to alternative interpretations depending on the reliability of the Cre drivers^6^. For instance, Zeisberg et al. have shown that at least 35% of all myofibroblasts could be derived from vascular endothelial cells labeled by *Tie2*^7^. However, an investigation by Kalluri and colleagues suggested, thanks to a more specific Cre driver (*Cdh5*-Cre), that the contribution of vascular endothelial cells to the myofibroblast pool was not more than 5%^8^. Humphreys et al., aided by the *Foxd1*-Cre reporter strain, demonstrated that nearly 100% of myofibroblasts could originate from pericytes or resident fibroblasts in two different models of renal fibrosis^9^. Myofibroblasts can be defined by a panel of marker genes, including *Col1a1*, *Col3a1*, *Acta2*, and, most importantly, *Postn*^10^. Indeed, *Postn* expression is induced dramatically in animal models of renal fibrosis, whereas *Postn* deletion attenuates renal fibrosis in mice^11–13^.

Myofibroblast transdifferentiation and renal fibrosis are programmed by a complex network of transcription factors^14^. Myocardin-related transcription factor A (MRTF-A), belonging to the myocardin family of transcriptional modulators, is considered a master regulator of organ fibrosis by promoting myofibroblast maturation^15^. Previous studies, largely relying on germline MRTF-A knockout (KO) mice, have provided compelling evidence that MRTF-A is essential for renal fibrosis in diabetic nephropathy^16^ and obstructive nephropathy^17^. Despite these observations that establish a pivotal role for MRTF-A in renal fibrosis, several outstanding questions remain. The present study sought to answer two questions: 1) whether myofibroblast-restricted deletion of MRTF-A is sufficient to dampen renal fibrosis and 2) which downstream target(s) of MRTF-A in renal fibroblasts might mediate the profibrogenic effects of MRTF-A. Our data show that MRTF-A deletion in the myofibroblast lineage is sufficient to improve renal fibrosis. MRTF-A likely contributes to renal fibrosis by activating a Zeb1–IRF9 transcriptional cascade.

## Methods

### Animal

All animal protocols were reviewed and approved by the intramural Ethics Committee on Humane Treatment of Laboratory Animals of Nanjing Medical University. The mice were kept in an SPF environment with 12 h light/dark cycles and libitum access to food and water. *Mrtfa*^f/f^ mice^18,19^ were crossed with*Postn*-Cre^ERT2^ mice^20^ to generate myofibroblast-specific MRTF-A knockout mice. Renal fibrosis was induced in 8-week-old male mice by either the unilateral ureteral obstruction (UUO) procedure or the ischemia‒reperfusion procedure as previously described^21–23^. To induce diabetic nephropathy, the mice were injected with a single dose of streptozotocin (STZ, 200 mg/kg) followed by feeding with a high-fat diet (HFD) for 14 weeks as previously described^24–26^.

### Cell culture, plasmids, and transient transfection

Human embryonic kidney cells (HEK293) were maintained in DMEM supplemented with 10% fetal bovine serum (FBS, HyClone) as previously described^27,28^. Primary murine renal fibroblasts were isolated and cultured as previously described^29^. The*Zeb1* promoter–luciferase construct was generated by amplifying genomic DNA spanning the proximal promoter and the first exon of the Zeb1 gene (-1000/+100) and ligating it into a pGL3-basic vector (Promega). The MRTF-A^30^ and Zeb1^31^ expression vectors have been previously described. Truncation mutants were made using a QuikChange kit (Thermo Fisher Scientific, Waltham, MA, United States) and were verified by direct sequencing. Small interfering RNAs were purchased from Dharmacon. Transient transfections were performed with Lipofectamine 2000. Luciferase activities were assayed 24-48 hours after transfection using a luciferase reporter assay system (Promega) as previously described^32^.

### RNA isolation and real-time PCR

RNA was extracted with the RNeasy RNA isolation kit (Qiagen) as previously described^33,34^. Reverse transcriptase reactions were performed using a SuperScript First-strand Synthesis System (Invitrogen). Real-time PCRs were performed on an ABI Prism 7500 system. The primers are listed in Supplementary Table I. Ct values of target genes were normalized to the Ct values of housekeeping control genes (18 s, 5’-CGCGGTTCTATTTTGTTGGT-3’ and 5’-TCGTCTTCGAAACTCCGACT-3’ for both human and mouse genes) using the ΔΔCt method and are expressed as relative mRNA expression levels compared to the control level, which was arbitrarily set as 1.

### Protein extraction and Western blot

Whole-cell lysates were obtained by resuspending cell pellets in RIPA buffer (50 mMTris pH 7.4, 150 mMNaCl, 1% Triton X-100) with freshly added protease and phosphatase inhibitors (Roche) as previously described^35^. The antibodies used for Western blotting are listed in Supplementary Table II. For densitometrical quantification, the densities of target proteins were normalized to those of β-actin. Data are expressed as relative protein levels compared to the control group level, which was arbitrarily set as 1.

### Chromatin immunoprecipitation (ChIP)

Chromatin immunoprecipitation (ChIP) assays were performed essentially as described before^36,37^. Chromatin was cross-linked with 1% formaldehyde for 8 min at room temperature and then sequentially washed with ice-cold phosphate-buffered saline, Solution I (10 mM HEPES, pH 7.5, 10 mM EDTA, 0.5 mM EGTA, 0.75% Triton X-100), and Solution II (10 mM HEPES, pH 7.5, 200 mMNaCl, 1 mM EDTA, 0.5 mM EGTA). Cells were incubated in lysis buffer (150 mMNaCl, 25 mMTris pH 7.5, 1% Triton X-100, 0.1% SDS, 0.5% deoxycholate) supplemented with a protease inhibitor tablet. DNA was fragmented into 500 bp pieces using a Branson 250 sonicator. Aliquots of lysates containing 100 μg of protein were used for each immunoprecipitation reaction with the indicated antibodies followed by adsorption ontoprotein A/G PLUS-agarose beads (Santa Cruz Biotechnology). Precipitated DNA‒protein complexes were washed sequentially with RIPA buffer (50 mMTris, pH 8.0, 150 mMNaCl, 0.1% SDS, 0.5% deoxycholate, 1% Nonidet P-40, 1 mM EDTA), high salt buffer (50 mMTris, pH 8.0, 500 mMNaCl, 0.1% SDS, 0.5% deoxycholate, 1% Nonidet P-40, 1 mM EDTA), LiCl buffer (50 mMTris, pH 8.0, 250 mMLiCl, 0.1% SDS, 0.5% deoxycholate, 1% Nonidet P-40, 1 mM EDTA), and TE buffer (10 mMTris, 1 mM EDTA pH 8.0). DNA‒protein cross-linking was reversed by heating the samples to 65 °C overnight. Proteins were digested with proteinase K (Sigma), and DNA was phenol/chloroform-extracted and precipitated by 100% ethanol. Precipitated genomic DNA was amplified by real-time PCR using the primers listed in online Supplementary Table I. A total of 10% of the starting material was also included as the input.

### Immunofluorescence staining

Paraffin sections were blocked with 5% BSA and incubated with the indicated primary antibodies overnight. After several washes with PBS, the slides were incubated with FITC-labeled secondary antibodies (Jackson) for 30 min. DAPI (Sigma) was added and incubated for 5 min prior to observation. Immunofluorescence was visualized under a confocal microscope (LSM 710, Zeiss).

### Histology

Histological analyses were performed essentially as described before^37–39^. Pictures were taken using an Olympus IX-70 microscope. Quantifications were performed with ImageJ. For each mouse, at least three slides were stained, and at least five different fields were analyzed in each slide.

### RNA sequencing and data analysis

RNA-seq was performed as previously described^40–42^. Total RNA was extracted using TRIzol reagent according to the manufacturer’s protocol. RNA purity and quantity were evaluated using a NanoDrop 2000 spectrophotometer (Thermo Scientific, USA). RNA integrity was assessed using the Agilent 2100 Bioanalyzer (Agilent Technologies, Santa Clara, CA, USA). Then, the libraries were constructed using the TruSeq Stranded mRNA LT Sample Prep Kit (Illumina, San Diego, CA, USA) according to the manufacturer’s instructions and were sequenced on an Illumina HiSeq X Ten platform, and 150 bp paired-end reads were generated. Raw data (raw reads) in fastq format were first processed using Trimmomatic, and low-quality reads were removed to leave only clean reads. The clean reads were mapped to the mouse genome (Mus_musculus.GRCm38.99) using HISAT2. The FPKM of each gene was calculated using Cufflinks, and the read counts of each gene were obtained by HTSeqcount. Differential expression analysis was performed using the DESeq (2012) R package. A P value < 0.05 and a fold change >2 or <0.5 was set as the threshold for significantly differential expression. Hierarchical cluster analysis of differentially expressed genes (DEGs) was performed to demonstrate the expression pattern of genes in different groups and samples. GO enrichment and KEGG pathway enrichment analyses of DEGs were performed using R based on the hypergeometric distribution.

### Statistical analysis

For comparisons between two groups, a two-tailed t test was performed. For comparison among three or more groups, one-way ANOVA or two-way ANOVA with the post-hoc Tukeytestwere performed in SPSS. The assumptions of normality were checked using the Shapiro‒Wilk test, and equal variance was checked using Levene’s test; both were satisfied. *p* values smaller than 0.05 were considered statistically significant (*). All in vitro experiments were repeated at least three times, and three replicates were estimated to provide 80% power.

## Results

### Myofibroblast-specific MRTF-A deletion attenuates renal fibrosis in mice

**Fig. 1:**
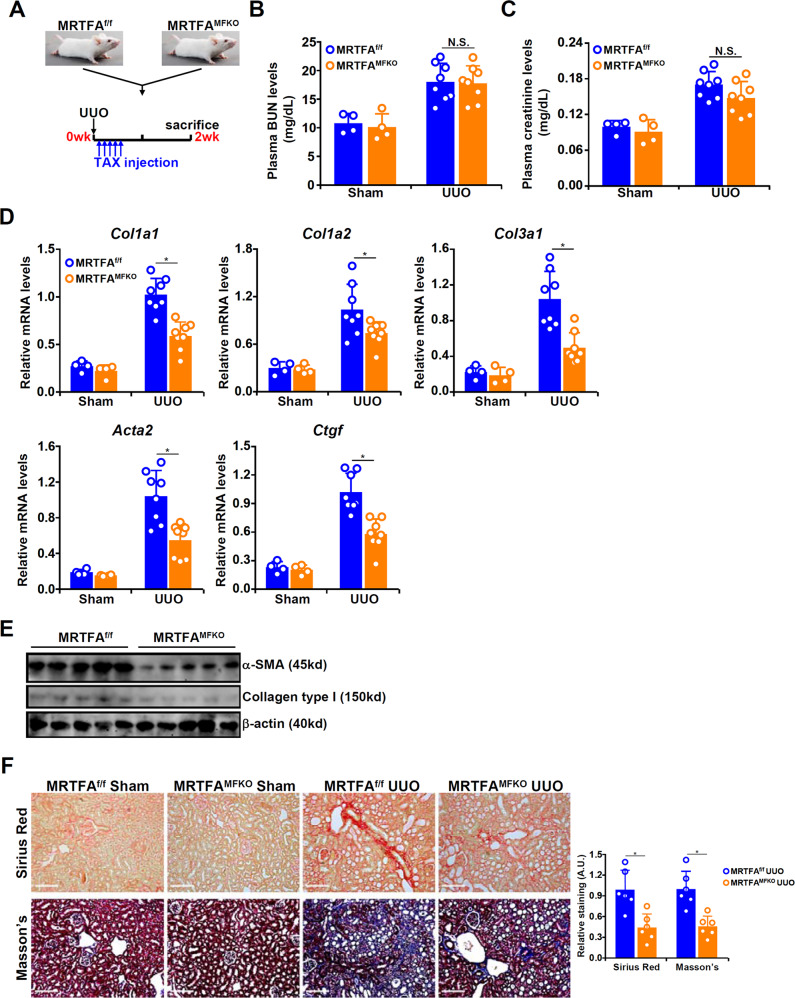
Myofibroblast-specific MRTF-A deletion attenuates renal fibrosis in mice. MRTF-A^f/f^ mice and MRTF-A^MFKO^ mice were subjected to the UUO procedure. **A** Scheme of the experimental protocol. **B** Plasma creatinine levels. **C** Plasma BUN levels. **D, E** Pro-fibrogenic gene expression levels were examined by qPCR and Western blotting. **F** Picrosirius red and Masson’s trichrome staining. *N* = 4-8 mice for each group.

Myofibroblast-specific MRTF-A knockout mice (MRTFA^MFKO^) and control mice (MRTFA^f/f^) were subjected to unilateral ureteral obstruction (UUO) followed by tamoxifen injection for 5 consecutive days; the mice were sacrificed 2 weeks after the UUO procedure (Fig. 1A). Immunofluorescence staining showed that fewer α-SMA^+^/MRTF-A^+^ cells were detected in MRTF-A^MFKO^ mice than in MRTF-A^f/f^ mice (Supplementary Fig. 1). The UUO procedure elicited similar increases in plasma BUN levels (Fig. 1B) and creatinine levels (Fig. 1C) in the MRTFA^MFKO^ mice and the MRTFA^f/f^ mice, suggesting that MRTF-A deficiency in myofibroblasts likely did not affect the glomerular filtration function. Quantitative PCR showed that the expression levels of profibrogenic genes, including *Col1a1*, *Col1a2*, *Col3a1*, *Acta2*, and *Ctgf*, were downregulated in the MRTFA^MFKO^ mice compared to the MRTFA^f/f^ mice (Fig. 1D, E). Picrosirius red staining and Masson’s trichrome staining confirmed that renal fibrosis was less extensive in the MRTFA^MFKO^ mice than in the MRTFA^f/f^ mice (Fig. 1F). Of note, immunohistochemical staining showed a reduction in both the a-SMA^+^ area and a reduction in the FSP1^+^ area in the MRTF-A^MFKO^ mice compared with the MRTF-A^f/f^ mice, indicating that MRTF-A deficiency might lead to attenuated expansion of fibroblasts/myofibroblasts (Supplementary Fig. 2). Similar observations were made in an alternative model of renal fibrosis induced by ischemia-reperfusion injury (Supplementary Fig. 3). Together, these data suggest that myofibroblast-specific MRTF-A deletion might be sufficient to influence renal fibrosis.

### RNA-seq reveals Zeb1 as a target of MRTF-A

**Fig. 2:**
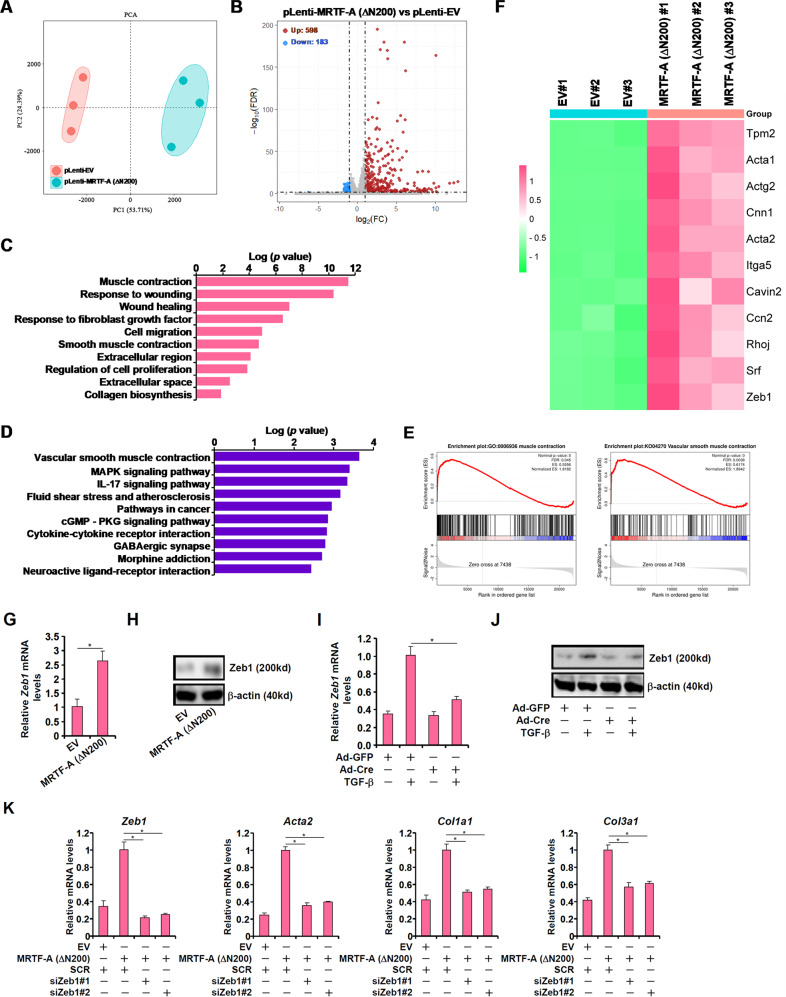
RNA-seq reveals Zeb1 as a target of MRTF-A. **A-F** Primary murine renal fibroblasts were transduced with lentivirus carrying an MRTF-A expression vector (ΔN200) or empty vector (EV). RNA-seq was performed as described in the Methods. PCA plot (**A**). Volcano plot (**B**). GO analysis (**C**). KEGG analysis (**D**). GSEA (**E**). Heatmap of differentially expressed genes (**F**). **G, H** Primary murine renal fibroblasts were transduced with lentivirus carrying an MRTF-A expression vector (ΔN200) or empty vector (EV). Zeb1 expression was examined by qPCR and Western blotting. **I, J** Primary murine renal fibroblasts were isolated from MRTF-A^f/f^ mice and transduced with adenovirus carrying Cre or GFP followed by treatment with TGF-β (2 ng/ml) for 24 h. Zeb1 expression was examined by qPCR and Western blot. **K** Primary murine renal fibroblasts were transduced with MRTF-A ΔN200 followed by transfection with the indicated siRNAs. Myofibroblast marker genes were examined by qPCR.

To identify transcriptional targets downstream of MRTF-A that could mediate its impact on FMyT and renal fibrosis, the following experiment was performed. An MRTF-A mutant (ΔN200), which resides in the nucleus by default and is thus considered constitutively active^43^, was packaged into lentivirus to transduce primary murine renal fibroblasts. Compared to the empty vector (EV), MRTF-A ΔN200 transduced into cells significantly elevated the levels of myofibroblast marker genes measured by qPCR (Supplementary Fig. 4a), stimulated cell proliferation (Supplementary Fig. 4b), and enhanced cell contraction (Supplementary Fig. 4c), all indicative of at least partial FMyT taking place. This was followed by RNA-seq analysis to evaluate changes in the cellular transcriptome (Fig. 2A). Overexpression of MRTF-A ΔN200 resulted in far more genes being upregulated (598) than downregulated (183), consistent with its role as a transcriptional activator (Fig. 2B). GO analysis (Fig. 2C), KEGG analysis (Fig. 2D), and gene set enrichment analysis (Fig. 2E) of differentially expressed genes all indicated that multiple pathways involved in FMyT, including the acquisition of the contractile phenotype, production of extracellular matrix proteins, and cell migration, were influenced by MRTF-A overexpression. Among the most significantly altered geneswere well-characterized MRTF-A target genes, including contractile genes (e.g., *Acta2* and*Cnn1*), extracellular matrix components (e.g., *Ccn2*), and regulators of cytoskeletal remodeling (e.g., *Rhoj*); Zeb1 was the only top-ranked transcription factor upregulated by MRTF-A overexpression (Fig. 2F). We focused on Zeb1 for the remainder of the study. qPCR (Fig. 2G) and Western blotting (Fig. 2H) confirmed that MRTF-A overexpression increased Zeb1 levels. In contrast, TGF-β-induced Zeb1 expression was attenuated by MRTF-A deletion in renal fibroblasts (Fig. 2I, J). Finally, the induction of myofibroblast marker genes by MRTF-A overexpression was dampened by Zeb1 depletion (Fig. 2K), suggesting that Zeb1 might be a crucial mediator of MRTF-A in programming fibroblast–myofibroblast transition.

### MRTF-A interacts with TEAD1 to regulate Zeb1 transcription

**Fig. 3:**
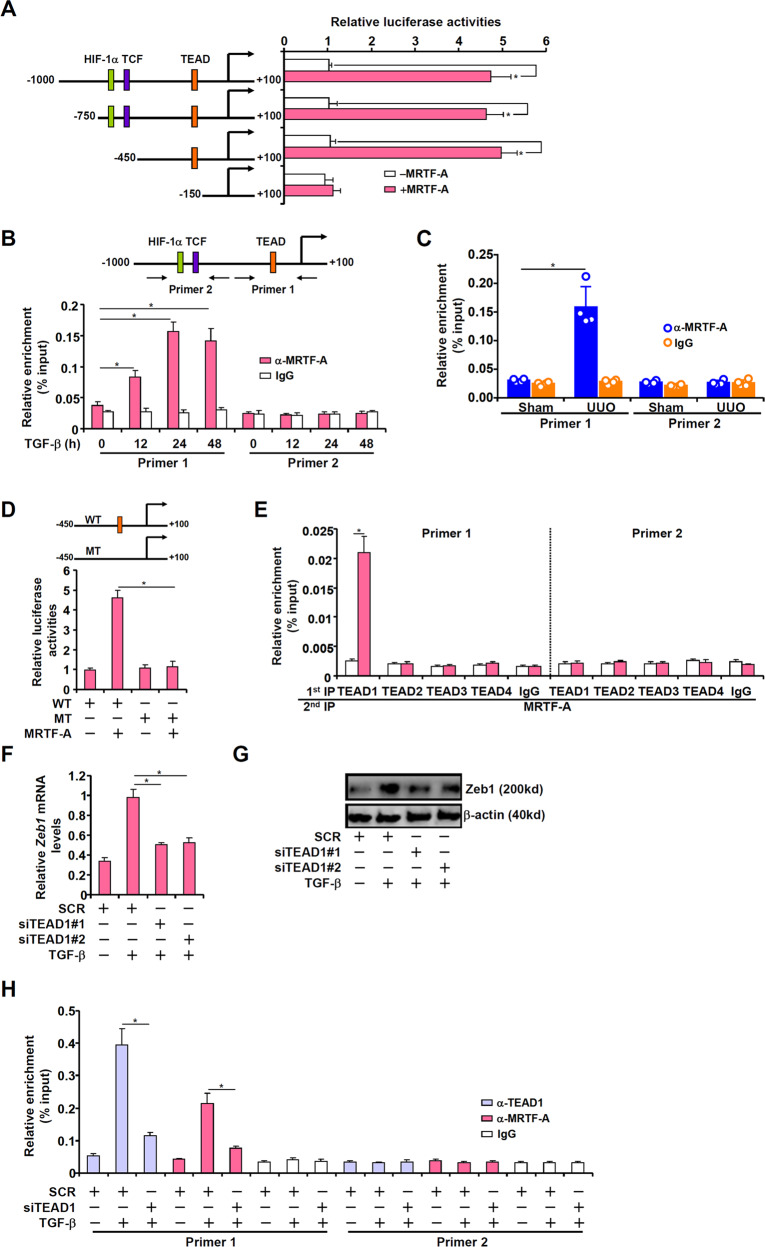
MRTF-A interacts with TEAD1 to regulate Zeb1 transcription. **A** Truncated Zeb1 promoter–luciferase constructs were transfected into HEK293 cells with or without MRTF-A. Luciferase activities were normalized by protein concentration and GFP fluorescence. **B** Primary renal fibroblasts were treated with TGF-β (2 ng/ml) and harvested at the indicated time points. ChIP assays were performed with anti-MRTF-A or IgG. **C** C57/B6 mice were subjected to the UUO procedure or the sham procedure as described in the Methods. ChIP assays were performed with kidney tissues. **D** Wild-type and mutated Zeb1 promoter-luciferase constructs were transfected into HEK293 cells with or without MRTF-A. Luciferase activities were normalized by protein concentration and GFP fluorescence. **E** Primary renal fibroblasts were treated with TGF-β (2 ng/ml) for 24 h. Re-ChIP assays were performed with the indicated antibodies. **F–H** Primary renal fibroblasts were transfected with siRNA targeting TEAD1 or scrambled siRNA (SCR) followed by treatment with TGF-β (2 ng/ml) for 24 h. Zeb1 expression was examined by qPCR and Western blot. ChIP assays were performed with the indicated antibodies.

We next investigated the mechanism whereby MRTF-A regulates Zeb1 expression. To this end, Zeb1 promoter–luciferase constructs with serial deletions were transfected into HEK293 cells with or without MRTF-A (Fig. 3A). MRTF-A stimulated Zeb1 promoter activity unless a region between −450 and −150 relative to the transcription start site, which contains a TEAD consensus motif, was removed (Fig. 3A). Chromatin immunoprecipitation (ChIP) provided corroborating evidence that MRTF-A was recruited to the TEAD region, but not to the more distal region, of the Zeb1 promoter by TGF-β treatment in renal fibroblasts (Fig. 3B). In addition, a stronger association of MRTF-A with the Zeb1 promoter was detected in the kidney tissues from the mice subjected to the UUO procedure (Fig. 3C) and the mice subjected to the ischemia‒reperfusion procedure (Supplementary Fig. 5) than from those subjected to the sham procedure. Mutation of the TEAD motif, however, desensitized the Zeb1 promoter to MRTF-A overexpression, again confirming that MRTF-A might rely on TEAD to be recruited to the Zeb1 promoter to activate transcription (Fig. 3D).

Four TEAD proteins have been identified in mammals^44^. Re-ChIP assays indicated that TEAD1, but not TEAD2, -3, or -4, could be assembled into a complex with MRTF-A on the Zeb1 promoter when renal fibroblasts were treated with TGF-β (Fig. 3E). Further, when TEAD1 was depleted with siRNA (see Supplementary Fig. 6 for knockdown efficiencies), Zeb1 wasdownregulated with a concomitant loss of MRTF-A binding to the Zeb1 promoter (Fig. 3F–H). Together, these data demonstrate that MRTF-A interacts with TEAD1 to regulate Zeb1 transcription.

### Zeb1 regulates FMyTin vitro and renal fibrosis in vivo

**Fig. 4:**
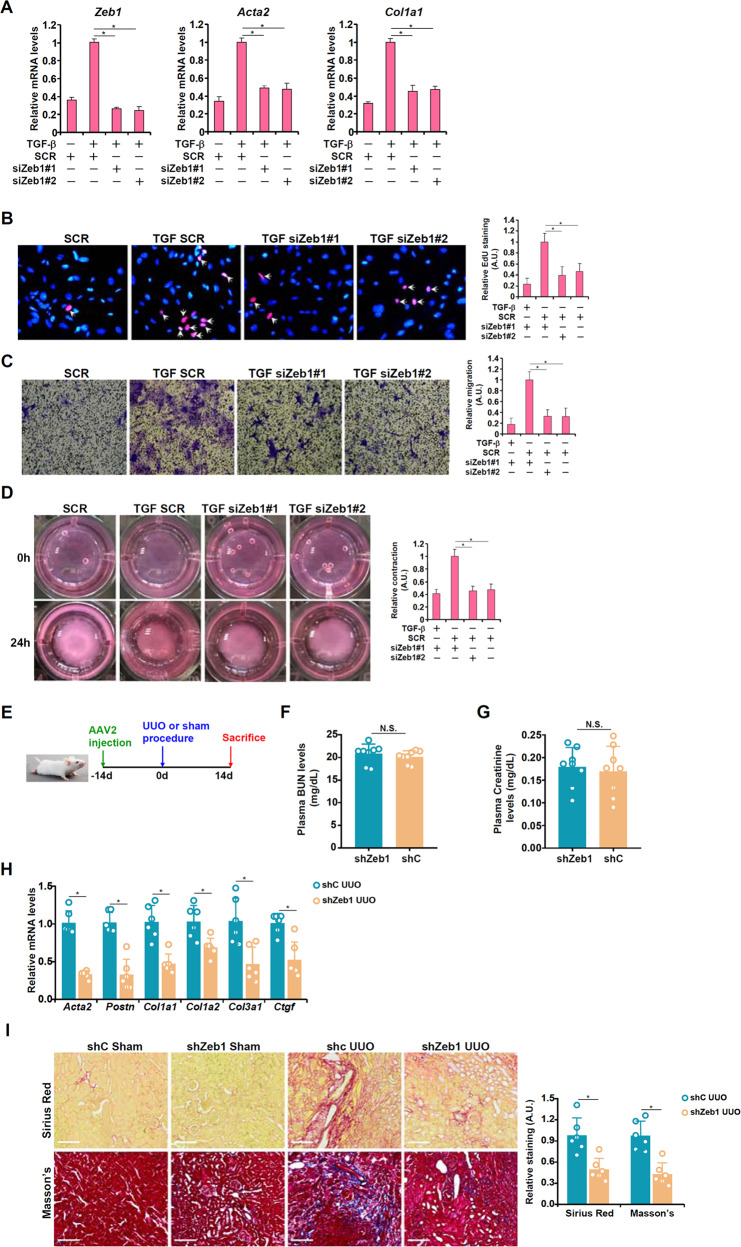
Zeb1 regulates FMyTin vitro and renal fibrosis in vivo. **A–D** Primary murine renal fibroblasts were transfected with siRNA targeting Zeb1 or scrambled siRNA (SCR) followed by treatment with TGF-β (2 ng/ml) for 24 h. Myofibroblast marker gene expression was examined by qPCR (**A**). Cell proliferation and migration were examined by EdU incorporation (**B**) and Transwell assays (**C**), respectively. **D** Collagen contraction assay. **E–I** C57/B6 mice were injected via tail vein AAV2 carrying shRNA targeting Zeb1 (shZeb1) or control shRNA (shC) followed by the UUO procedure to induce renal fibrosis. The mice were sacrificed 2w after the surgery. (**E**) Scheme of the experimental protocol. **F** Plasma creatinine levels. **G** Plasma BUN levels. **H** Pro-fibrogenic gene expression levels were examined by qPCR. **I** Picrosirius red and Masson’s trichrome staining. *N* = 6-8 mice for each group.

Next, the role of Zeb1 in fibroblast–myofibroblast transition and renal fibrosis was determined. In the first set of experiments,Zeb1 depletion suppressed TGF-β-induced myofibroblast marker gene expression (Fig. 4A), proliferation (Fig. 4B), migration (Fig. 4C), and muscle-like contraction (Fig. 4D) in renal fibroblasts, as measured by qPCR, EdU incorporation, Boyden chamber Transwell assay, and collagen contraction assay, respectively. These observations suggest that Zeb1 might be indispensable for TGF-β-induced fibroblast–myofibroblast transition in vitro.

A short hairpin RNA (shRNA) sequence targeting Zeb1 (shZeb1) was placed downstream of a myofibroblast-specific promoter (*Postn*) and delivered into mice via AAV2^45^. Quantitative PCR verified that Zeb1 expression was downregulated by shZeb1 in the fibroblast fraction but not in the nonfibroblast fraction in the kidneys (Supplementary Fig. 7). Efficient knockdown was further verified by immunofluorescence staining, which showed that fewer α-SMA^+^/ZEB1^+^ cells were detected in the shZeb1 mice than in the shC mice (Supplementary Fig. 8).The mice were then subjected to the UUO procedure to induce renal fibrosis (Fig. 4E). Measurements of plasma BUN (Fig. 4F) and plasma creatinine (Fig. 4G) indicated that glomerular infiltration function was comparable between mice injected with shZeb1 AAV2 and mice injected with shC AAV2. The expression levels of profibrogenic genes, however, were collectively downregulated in the shZeb1 mice compared to the shC mice (Fig. 4H). Picrosirius red staining and Masson’s trichrome staining both indicated diminished levels of renal fibrosis in the shZeb1 mice compared to the shC mice (Fig. 4I). Immunohistochemical staining showed a reduction in both a-SMA^+^ area and FSP1^+^ area in shZeb1 mice compared with shC mice, indicating that Zeb1, like MRTF-A, might contribute to the expansion of fibroblasts/myofibroblasts (Supplementary Fig. 9). Similar observations were made in a second model, of renal ischemia‒reperfusion injury (Supplementary Fig. 10), suggesting that Zeb1 depletion in myofibroblasts could indeed ameliorate renal fibrosis. In a third model, of renal fibrosis induced by diabetic nephropathy,created by injecting mice with STZ followed by HFD feeding, Zeb1 depletion did not alter body weight (Supplementary Fig. 11a) or plasma glucose levels (Supplementary Fig. 11b). However, Zeb1 depletion improved renal function, as determined by plasma BUN (Supplementary Fig. 11c) and creatinine (Supplementary Fig. 11d) levels, and mitigated renal fibrosis, as determined by qPCR measurements of profibrogenic gene expression (Supplementary Fig. 11e) and PSR/Masson’s staining (Supplementary Fig. 11f).

### IRF9 is a novel target of Zeb1

**Fig. 5:**
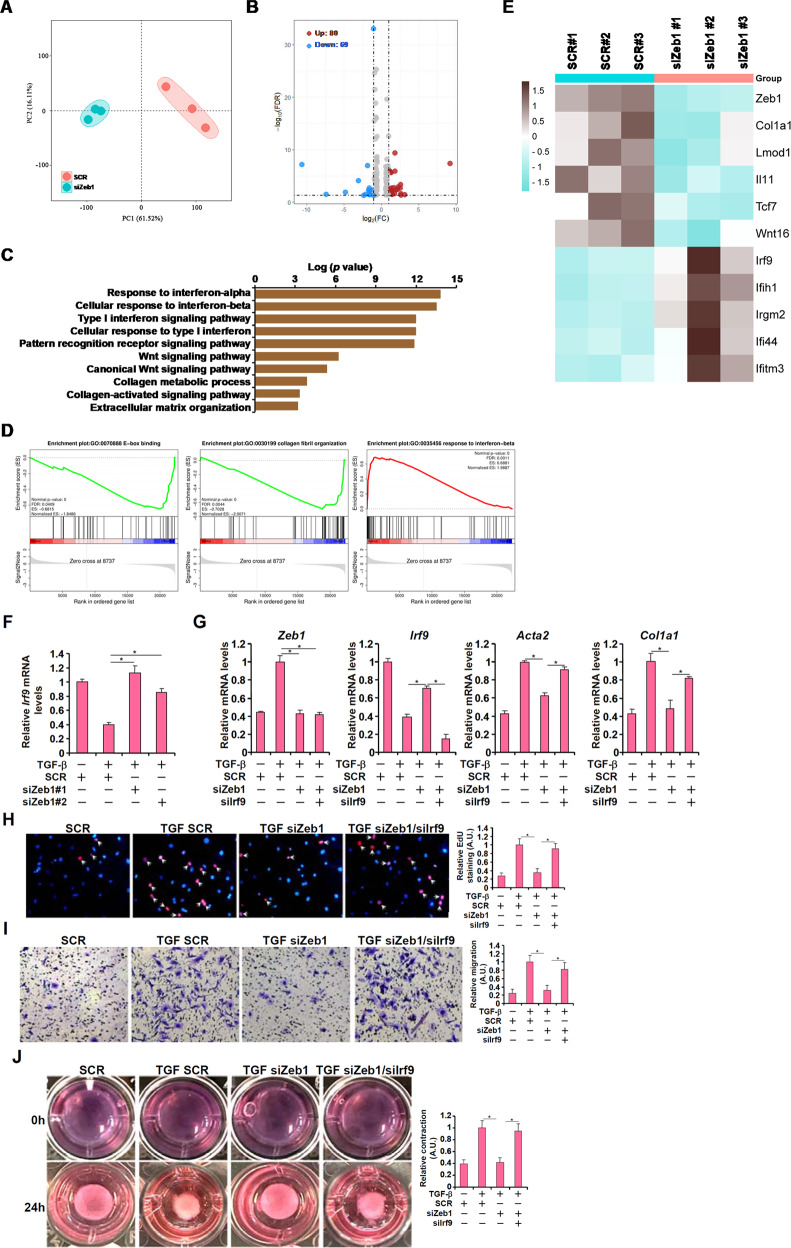
IRF9 is a novel target of Zeb1. **A–E** Primary murine renal fibroblasts were transfected with siRNA targeting Zeb1 or scrambled siRNA (SCR) followed by treatment with TGF-β (2 ng/ml) for 24 h. RNA-seq was performed as described in the Methods. PCA plot (**A**). Volcano plot (**B**). GO analysis (**C**). GSEA (**D**). Heatmap of differentially expressed genes (**E**). **F** Primary murine renal fibroblasts were transfected with siRNA targeting Zeb1 or scrambled siRNA (SCR) followed by treatment with TGF-β (2 ng/ml) for 24 h. IRF9 expression was examined by qPCR. **G–J** Primary murine renal fibroblasts were transfected with the indicated siRNAs followed by treatment with TGF-β (2 ng/ml) for 24 h. Myofibroblast marker gene expression was examined by qPCR (**G**). Cell proliferation and migration were examined by EdU incorporation (**H**) and Transwell assays (**I**), respectively. **J** Collagen contraction assay.

To probe the mechanism whereby Zeb1 might influence FMyT and promote renal fibrosis, RNA-seq analysis was performed in primary renal fibroblasts transfected with siRNA targeting Zeb1 or scrambled siRNA followed by treatment with TGF-β (Fig. 5A). Using a 1.5-foldchange and p < 0.0.5 as thresholds, a total of 149 differentially expressed genes were identified, with 80 genes being upregulated and 69 genes being downregulated by Zeb1 knockdown (Fig. 5B). GO analysis indicated that several FMyT-related pathways, including the Wnt pathway, were influenced by the absence of Zeb1 (Fig. 5C). Gene set enrichment analysis (GSEA) indicated that Zeb1 deficiency was negatively associated with global E-box binding and collagen fiber organization but positively correlated with the type I interferon response (Fig. 5D). Indeed, myofibroblast marker genes (*Col1a1*, *Lmod1*) and components of the Wnt pathway (*Tcf7*, *Wnt16*) were downregulated, whereas several IFN-inducible genes were upregulated by Zeb1 depletion (Fig. 5E). We focused on interferon response factor 9 (IRF9) because it is one of the upstream mediators of the interferon response.

Quantitative PCR data showed that IRF9 expression levels were downregulated during FMyTin vitro and in vivo (Supplementary Fig. 12). Zeb1 knockdown, however, completely abrogated the IRF9 downregulation by TGF-β in primary renal fibroblasts (Fig. 5F). Importantly, IRF9 knockdown rescued the defect in FMyT caused by Zeb1 depletion, as evidenced by myofibroblast marker gene expression (Fig. 5G), proliferation (Fig. 5H), migration (Fig. 5I), and muscle-like contraction (Fig. 5J).

### IRF9 is a novel regulator of TGF-β-induced fibroblast–myofibroblast transition

**Fig. 6:**
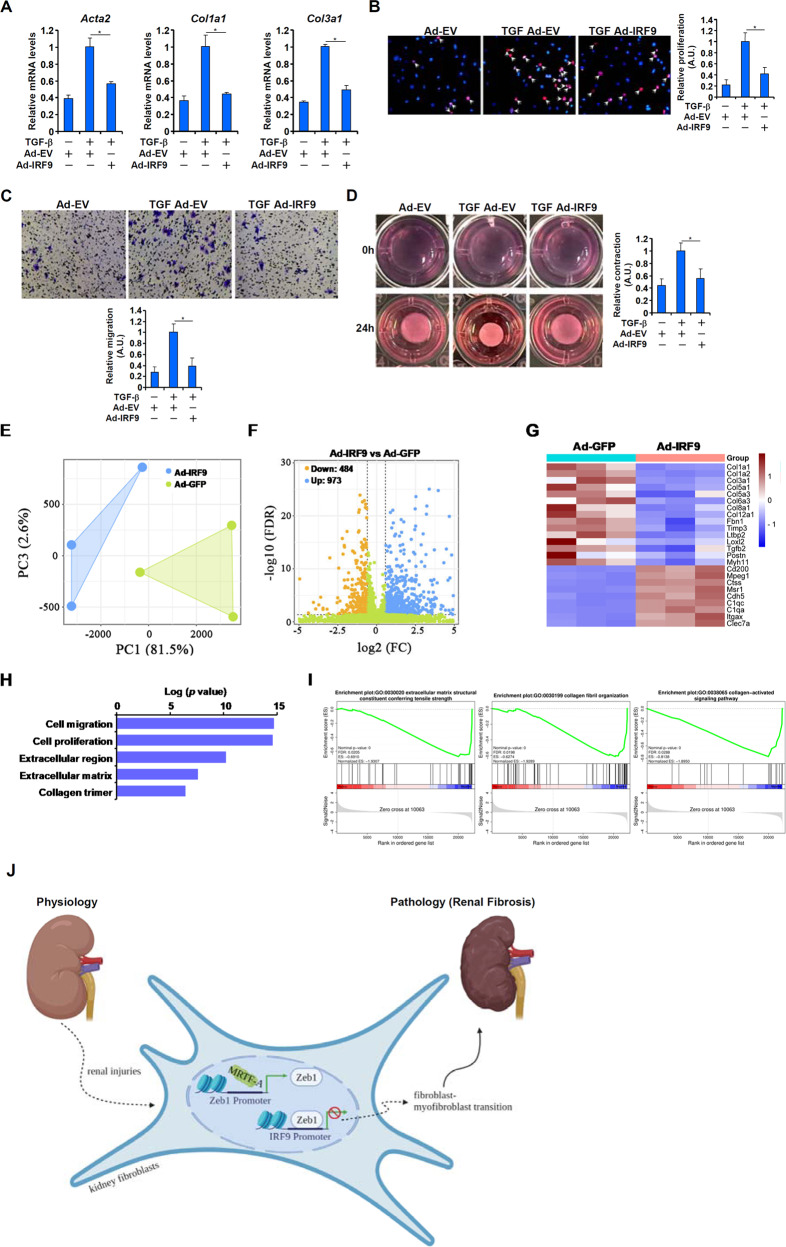
IRF9 is a novel regulator of TGF-β-induced fibroblast–myofibroblast transition. **A-D** Primary murine renal fibroblasts were transduced with adenovirus carrying an IRF9 vector (Ad-IRF9) or an empty vector (Ad-EV) followed by treatment with TGF-β (2 ng/ml) for 24 h. Myofibroblast marker gene expression was examined by qPCR (**A**). Cell proliferation and migration were examined by EdU incorporation (**B**) and Transwell assays (**C**), respectively. (d) Collagen contraction assay. **E–I** Primary murine renal fibroblasts were transduced with adenovirus carrying an IRF9 vector (Ad-IRF9) or an empty vector (Ad-EV)followed by treatment with TGF-β (2 ng/ml) for 24 h. RNA-seq was performed as described in the Methods. PCA plot (**E**). Volcano plot (**F**). GO analysis (**G**). GSEA (**H**). Heatmap of differentially expressed genes (**I**). **J** A schematic model.

Finally, we investigated the role of IRF9 in FMyT. Overexpression of IRF9 significantly antagonized TGF-β-induced FMyT, as evidenced by a reduction in myofibroblast marker gene expression (Fig. 6A), decreased cell proliferation (Fig. 6B), diminished cell migration (Fig. 6C), and suppression of cell contraction (Fig. 6D).

Next, RNA-seq was performed to evaluate whether IRF9 might contribute to FMyTthrougha transcriptomic mechanism. As shown in Fig. 6E, IRF9 overexpression significantly altered the gene expression profile of renal fibroblasts. In all, 484 genes were downregulated and 973 genes were upregulated (p < 0.0.5 and 1.5-foldchange) by IRF9 overexpression (Fig. 6F). Among the top differentially expressed genes, extracellular matrix proteins (e.g., collagens), profibrogenic growth factors (e.g., Tgfb2), contractile proteins (e.g., Myh11), and unique myofibroblast markers (e.g., Postn) were predominantly downregulated, whereas immune lineage markers (e.g., Cd200) and endothelial lineage markers (e.g., Cdh5) were upregulated (Fig. 6G). GO analysis (Fig. 6h) and gene set enrichment analysis (Fig. 6I) confirmed that IRF9 exerted a suppressive effect on FMyT by negatively regulating pathways involved in cell proliferation and ECM production.

## Discussion

Fibroblast–myofibroblast transition (FMyT) represents a key process that contributes to the pool of ECM-producing cells and promotes renal fibrosis^46^. In this report, we detail an MRTF-A–Zeb1–IRF9 transcriptional axis that mediates FMyT and renal fibrosis. Our data demonstrate that MRTF-A binds to the Zeb1 promoter and activates itstranscription, which in turn represses IRF9 transcription to drive a profibrogenic program (Fig. 6j). Previous studies have shown that systemic deficiency of MRTF-A leads to attenuation of renal fibrosis in mice^16,17^. MRTF-A deficiency also mitigates renal injury, as evidenced by reduced immune cell infiltration and improved glomerular infiltration. Early studies indicated that MRTF-A is ubiquitously expressed in adults^47^. We previously reported that myeloid-specific deletion of MRTF-A attenuates acute kidney injury (AKI) induced by LPS injection or ischemia‒reperfusion^48^. In the present study, myofibroblast-targeted deletion of MRTF-A (MRTF-A^MFKO^) mollifies renal fibrosis without influencing renal injuries. Collectively, these observations suggest that MRTF-A might play cell type-dependent roles in regulating renal injury and fibrosis: in acute phases, MRTF-A in immune cell lineages likely contributes to renal injury by promoting immune cell trafficking and ROS production; in late/repair phases, MRTF-A in myofibroblast lineages possibly contributes to renal fibrosis by promoting FMyT. Because myeloid cells have also been found to behave like myofibroblasts under certain circumstances and contribute to renal fibrosis^49^, these data argue for the benefit of targeting MRTF-A, regardless of its origin, in the treatment of renal fibrosis.

MRTF-A activity is influenced by cytoskeletal remodeling-dependent shuffling between the cytoplasm and the nucleus^50^. Here, we show that constitutively nucleus-bound MRTF-A is sufficient to drive FMyTin vitro. Further, RNA-seqexperiments employing this system revealed several previously unrecognized targets for MRTF-A, including Zeb1. Zeb1 knockdown abrogates the profibrogenic effect of MRTF-A in renal fibroblasts in vitro, whereas mice with myofibroblast-specific depletion of Zeb1 phenocopied the MRTF-A^MFKO^ mice in two different animal models, pointing to Zeb1 as a downstream mediator of MRTF-A in FMyT and renal fibrosis. Zeb1 belongs to the E-box binding family of transcriptional repressors that also include Snail, Slug, and Twist^51^. Sobue and colleagues have previously shown that MRTF-A can directly bind to the promoter region of Slug through itsinteraction with SMAD3 toactivate Slug transcription in renal tubular epithelial cells andpromote epithelial-mesenchymal transition (EMT)^52^. Although this finding is unlikely to be pathophysiologically significant in renal fibrosis because the epithelial lineage makes a negligible contribution to the myofibroblast pool in vivo, the similarity between EMT and FMyT corroborates our discoveries and confirmsthe pivotal role of MRTF-A in regulating/maintaining the myofibroblast phenotype. Germline Zeb1 deletion leads to perinatal lethality in mice, likely owing to defective osteochondrogenesis and/or hematopoiesis^53^. A floxed Zeb1 strain^54^ has been developed and could potentially be exploited to generate fibroblast/myofibroblast-specific Zeb1 knockout strains to further authenticate the role of Zeb1 in FMyTin vivo.

Transcriptomic analysis indicates that, in addition to several canonical profibrogenic pathways, Zeb1 might contribute to FMyT by influencing the cellular response to type I interferon. Traditionally, nonimmune cells (e.g., fibroblasts) predominantly produce IFN-β to influence the behavior of neighboring cells, a process mediated by the transcription factors STAT1 and IRF9^55^. The potential link between type I interferon signaling and FMyT is unclear at this point. Medley et al. recently showed that fibroblast-specific deletion of STAT1, driven by*Twist2*-Cre^ERT2^, promotes myofibroblast transition and enhances scarring in a skin punch model of wound healing^56^. Coincidently, STAT1 deletion leads to the downregulation of several interferon signaling genes (ISGs). In contrast, pharmaceutical targeting of STAT1 by an inhibitor of its upstream regulator JAK1 attenuates Dupuytren’s fibrosis^57^. Although the conclusiveness of the latter study may be questioned by itsusage of a nonspecific inhibitor, it is abundantly clear that further studies are warranted to delineate the mechanism whereby altered interferon signaling regulates FMyT and renal fibrosis.

Our data show that IRF9, downstream of Zeb1, antagonizes the TGF-β-induced FMyTin vitro. The underlying mechanism, however, remains obscure at this point. Transcriptomic examination revealed that IRF9 overexpression simultaneously dampens the expression of myofibroblast signature genes and stimulates the expression of immune/endothelial lineage signature genes. This observation raises an intriguing possibility that IRF9 might serve as a molecular switch dictating the programming/reprogramming of cell fate. Much evidence exists to support a role for various IRF family members in cell fate determination. For instance, IRF4 is considered a master regulator of T-cell fate decisions by coordinating the transcription of lineage-specific cytokines^58^. IRF2 functions as a blockade of pluripotency and transdifferentiation capacity in keratinocytes by modulating the chromatin landscape^59^. WhetherIRF9 possesses similar activities to regulate FMyT certainly deserves further attention.

Major limitations of the present study cast doubt on theinterpretationof the data and the overall translational potential of the findings. First, renal fibrosis develops and progresses to influence renal architecture and function over years, if not decades, in humans with chronic kidney diseases, a process that cannot be faithfully recapitulated by modeling in mice over the course of weeks. Indeed, manipulation of Zeb1 in myofibroblasts attenuated renal fibrosis without altering renal function in two acute models (UUO and IRI), whereas the same procedure both improved renal function and mitigated renal fibrosis in a more chronic model (diabetic nephropathy). This discrepancy suggests that more work is needed to determine the long-term effect of targeting the MRTF-A–Zeb1–IRF9 axis in CKD pathogenesis, ideally in humanized models. Second, proximal tubules represent the largest cell population in the adult kidneys^25^. Although tubular cells do not directly contribute to the pool of ECM-producing myofibroblasts, they nonetheless play key roles in renal fibrosis. Of note, tubular epithelial-targeted deletion of Snail^60^ or Twist^61^, both belonging to the same family of transcription factors as Zeb1, has been shown to dampen renal fibrosis and improve renal fibrosis in mice. It would be of great interest to determine whether manipulation of the MRTF-A–Zeb1–IRF9 axis in tubular cells would achieve similar effects.

In summary, our data reveal a previously unrecognized MRTF-A–Zeb1–IRF9 transcriptional cascade that contributes to renal fibrosis by regulating FMyT. Our data further support the notion that MRTF-A is the master regulator of myofibroblast fate. Additional studies are warranted to provide a stronger rationale for targeting this axis as part of novel pharmacotherapies against renal fibrosis.

## Supplementary information

online supplementary data

## Data Availability

The data that support the findings of this study are available upon reasonable request.
