# Supplementary material for: An MRTF-A–ZEB1–IRF9 axis contributes to fibroblast–myofibroblast transition and renal fibrosis
Source: Exp Mol Med. 2023 May 1;55(5):987–98. doi: 10.1038/s12276-023-00990-6 (PMC10238398; doi:10.1038/s12276-023-00990-6)
Supplement: Supplementary file 1 — online supplementary data [file 12276_2023_990_MOESM1_ESM.pdf]

Zhao QW et al: An MRTF-A–ZEB1–IRF9 axis contributes to fibroblast–myofibroblast transition and renal fibrosis

Online supplementary material

Supplementary figures: 12

Supplementary tables: 2

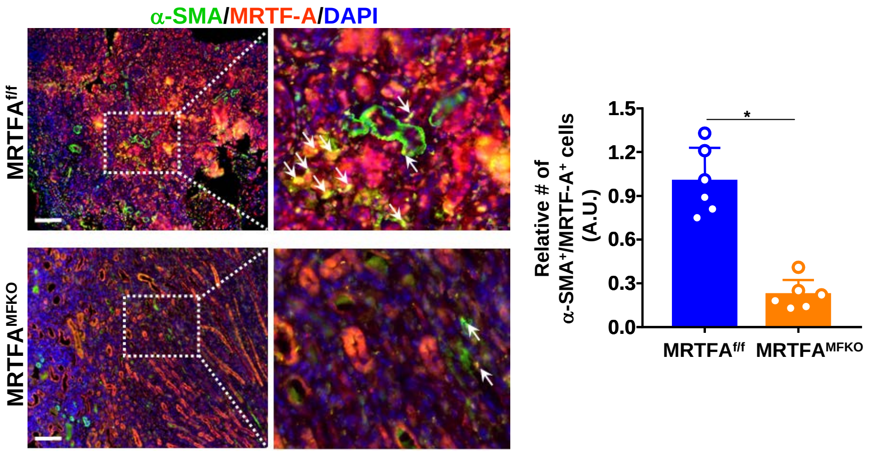

Supplementary Fig. 1: MRTF-A$^{f/f}$ mice and MRTF-A^{MFKO} mice were subjected to the UUO procedure. Immunofluorescence staining was performed with anti-α-SMA and anti-MRTF-A as described in Methods. N=6 mice for each group.

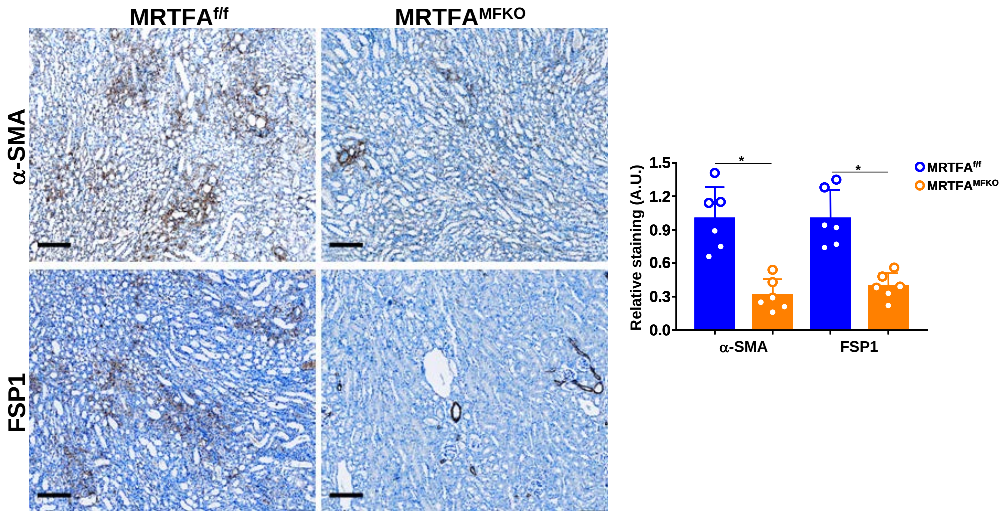

Supplementary Fig. 2: MRTF-A$^{f/f}$ mice and MRTF-A^{MFKO} mice were subjected to the UUO procedure. Immunohistochemical staining was performed with anti-α-SMA or anti-FSP1 as described in Methods. N=6 mice for each group.

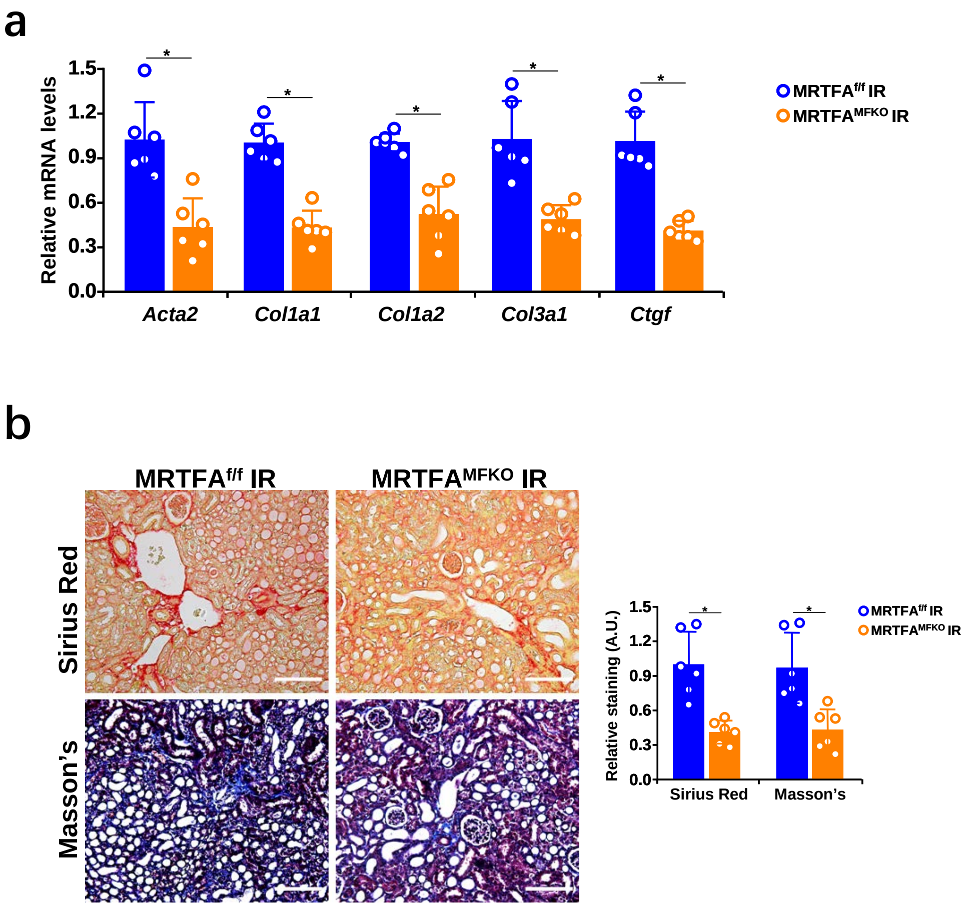

Supplementary Fig. 3: MRTF-A$^{f/f}$ mice and MRTF-A^{MFKO} mice were subjected to the ischemia-reperfusion procedure. (**a**) Pro-fibrogenic gene expression levels were examined by qPCR. (**b**) Picrosirius red and Masson's trichrome stainings. N=6 mice for each group.

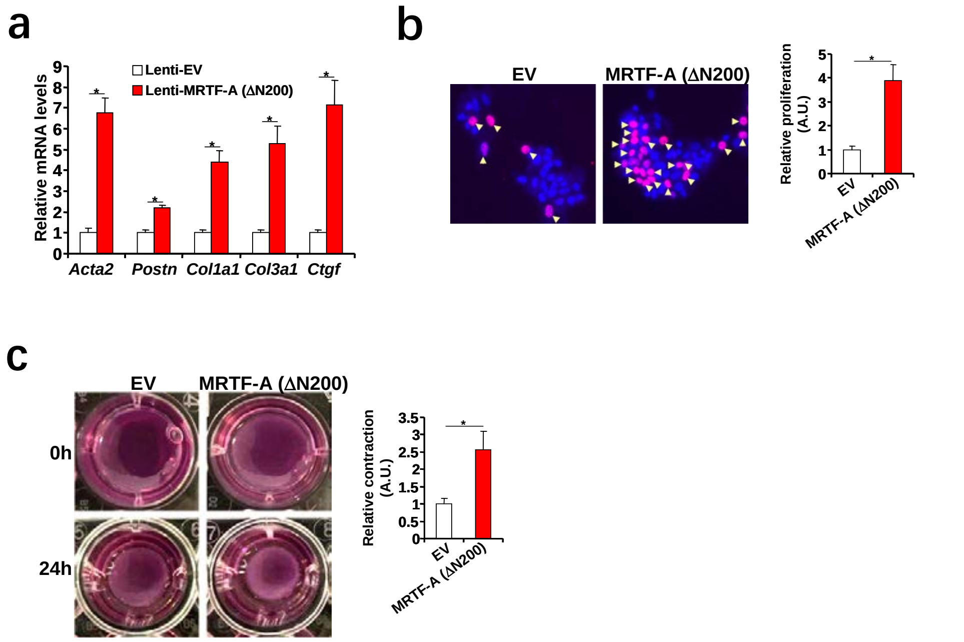

Supplementary Fig. 4: (**a-c**) Primary murine renal fibroblasts were transduced with lentivirus carrying an MRTF-A expression vector (ΔN200) or an empty vector (EV). Myofibroblast marker gene expression was examined by qPCR (a). Cell proliferation was examined by EdU incorporation (b). Collagen contraction assay (c).

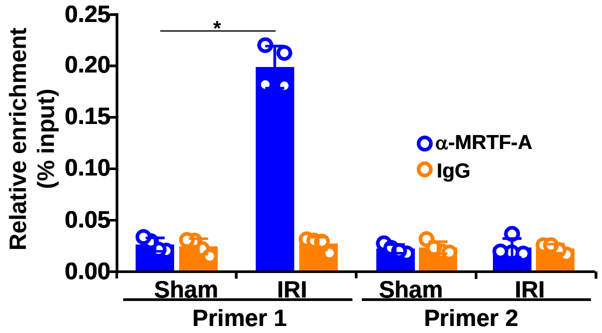

Supplementary Fig. 5: C57/B6 mice were subjected to the renal ischemia-reperfusion procedure or the sham procedure as described in Methods. ChIP assay was performed with kidney tissues.

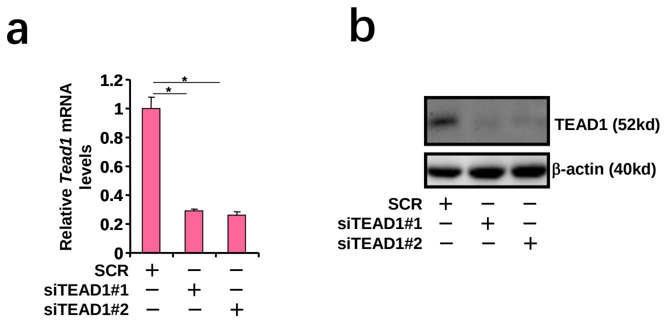

Supplementary Fig. 6: (**a, b**) Primary murine renal fibroblasts were transfected with indicated siRNAs. TEAD1 expression levels were examined by qPCR and Western blotting.

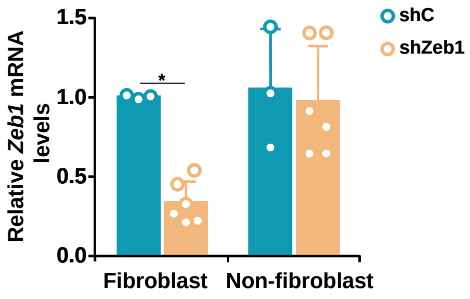

Supplementary Fig. 7: C57/B6 mice were injected via tail vein AAV2 carrying shRNA targeting Zeb1 (shZeb1) or control shRNA (shC). Fibroblast fractions and non-fibroblast fractions were isolated from the kidneys and Zeb1 expression was examined by qPCR.

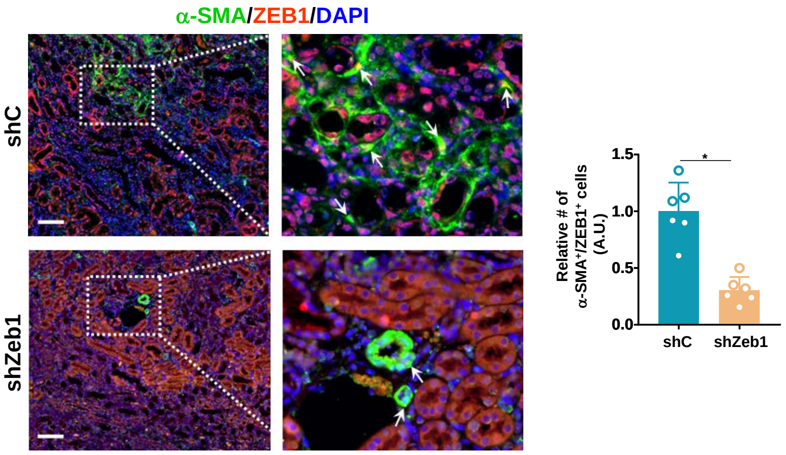

Supplementary Fig. 8: C57/B6 mice were injected via tail vein AAV2 carrying shRNA targeting Zeb1 (shZeb1) or control shRNA (shC) followed by the UUO procedure to induce renal fibrosis. Immunofluorescence staining was performed with anti-α-SMA and anti-ZEB1 as described in Methods. N=6 mice for each group.

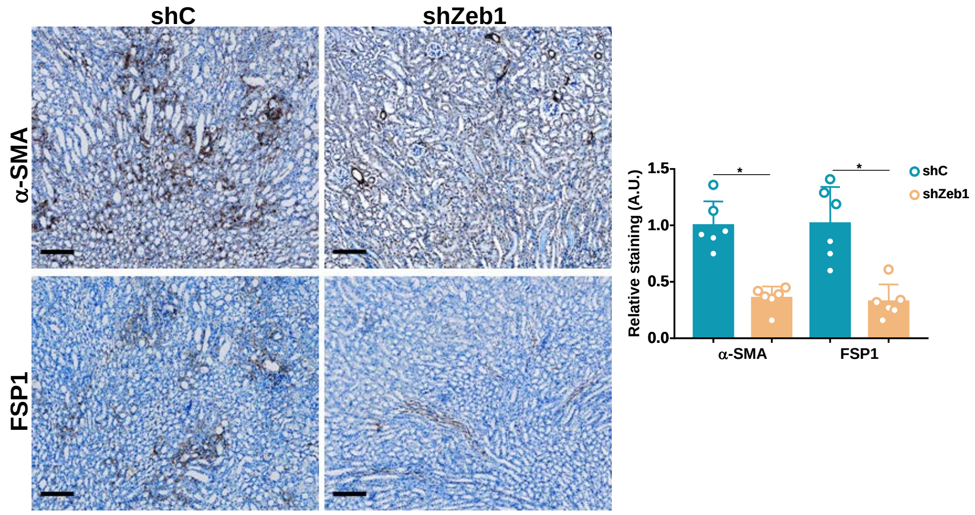

Supplementary Fig. 9: C57/B6 mice were injected via tail vein AAV2 carrying shRNA targeting Zeb1 (shZeb1) or control shRNA (shC) followed by the UUO procedure to induce renal fibrosis. Immunohistochemical staining was performed with anti-α-SMA or anti-FSP1 as described in Methods. N=6 mice for each group.

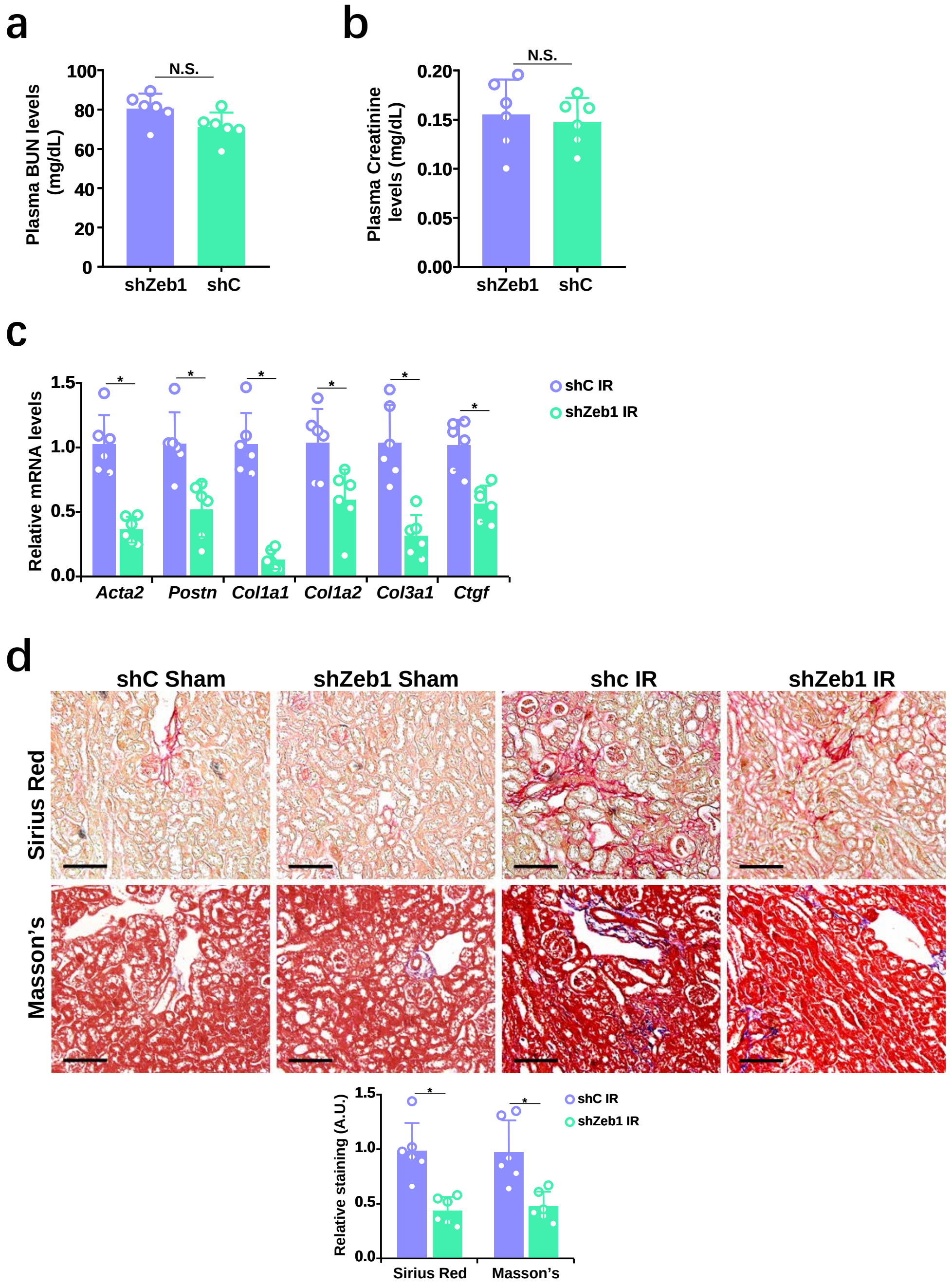

Supplementary Fig. 10: (**a-d**) C57/B6 mice were injected via tail vein AAV2 carrying shRNA targeting Zeb1 (shZeb1) or control shRNA (shC) followed by the ischemia-reperfusion procedure to induce renal fibrosis. The mice were sacrificed 2w after the surgery. (**a**) Plasma BUN levels. (**b**) Plasma creatinine levels. (**c**) Pro-fibrogenic gene expression levels were examined by qPCR. (**d**) Picrosirius red and Masson's trichrome stainings. N=6 mice for each group.

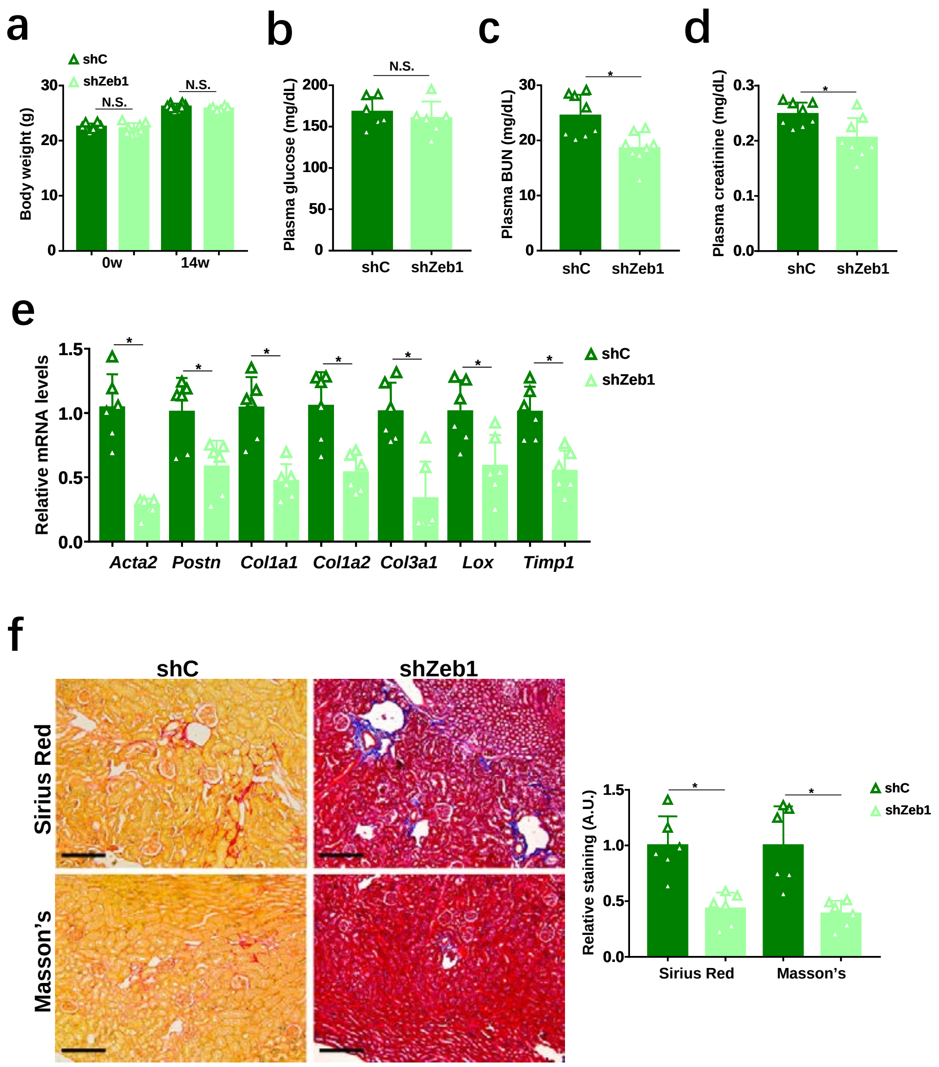

Supplementary Fig. 11: (**a-f**) C57/B6 mice were injected via tail vein AAV2 carrying shRNA targeting Zeb1 (shZeb1) or control shRNA (shC) followed by procedures to induce diabetic nephropathy as described in Methods. (**a**) Body weight. (**b**) Plasma glucose levels. (**c**) Plasma BUN levels. (**d**) Plasma creatinine levels. (**e**) Pro-fibrogenic gene expression levels were examined by qPCR. (**f**) Picrosirius red and Masson's trichrome stainings. N=6 mice for each group.

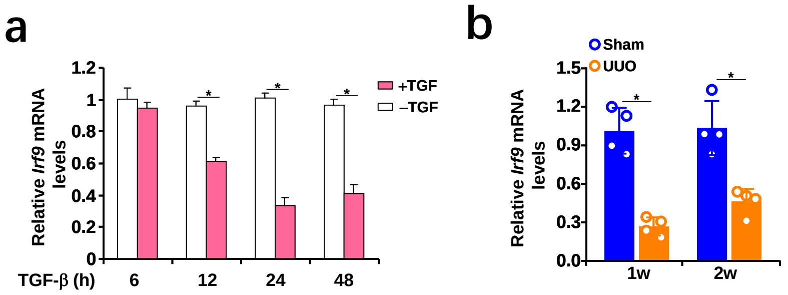

Supplementary Fig. 12: (**a**) Primary murine renal fibroblasts were treated with or without TGF-β and harvested at indicated time points. IRF9 expression was examined by qPCR. (**b**) C57/B6 mice were subjected to the UUO procedure or the sham procedure and sacrificed at indicated time points. Primary renal fibroblasts were isolated and IRF9 expression was examined by qPCR.

Supplementary Table I: QPCR Primers Sequences

**Gene**	**Forward primer**	**Reverse primer**
Mouse *Col1a1*	GCTCCTCTTAGGGGCCACT	ATTGGGGACCCTTAGGCCAT
Mouse *Col3a1*	CTGTAACATGGAAACTGGGGAAA	CCATAGCTGAACTGAAAACCACC
Mouse *Acta2*	CCCAGACATCAGGGAGTAATGG	TCTATCGGATACTTCAGCGTCA
Mouse *Col1a2*	TCGTGCCTAGCAACATGCC	TTTGTCAGAATACTGAGCAGCAA
Mouse *Ctgf*	GGCCTCTTCTGCGATTTCG	GCAGCTTGACCCTTCTCGG
Mouse *Zeb1*	ACCGCCGTCATTTATCCTGAG	CATCTGGTGTTCCGTTTTCATCA
Mouse *Irf9*	CCTCAGGCAAAGTACGCTG	GGGGTGTCCTATGTCCCCA
Mouse *Tead1*	GAGCGACTCGGCAGATAAGC	CCACACGGCGGATAGATAGC

Supplementary Table II: Antibody Information

Antigen	Vendor (catalog#)	Application
β-actin	Sigma (A2228)	IB
MRTF-A	Proteintech (21166-1)	IB, IF, ChIP
Zeb1	Proteintech (21544-1)	IB, IF, ChIP
TEAD1	Abcam (ab133533)	IB, ChIP
α-SMA	Abcam (ab7817)	IB, IHC, IF
Collagen type I	Rockland (600-401-103-01)	IB
FSP1	Proteintech (20886-1)	IHC
